# Supplementary material for: (2,6-Dimethylphenyl)arsonic Acid Induces Apoptosis through the Mitochondrial Pathway, Downregulates XIAP, and Overcomes Multidrug Resistance to Cytostatic Drugs in Leukemia and Lymphoma Cells In Vitro
Source: Int J Mol Sci. 2024 Apr 26;25(9):4713. doi: 10.3390/ijms25094713 (PMC11083614; doi:10.3390/ijms25094713)
Supplement: Supplementary file 1 [file ijms-25-04713-s001.zip › Supplementary Material.pdf]

Supplementary Material

Table S1. Drug resistant cell lines. Origin, primary resistances and co-resistances against common chemotherapeutic drugs as well as the proposed resistance mechanisms (if applicable) are depicted for the resistant cell lines used in this study. NA = not applicable/mechanism not known.

Cell line	Parental cell line	Primary resistance	Co-resistances	Suggested resistance mechanism
**BeKa**	Nalm-6	Vincristine	Colchicine, Daunorubicin, Doxorubicin, Epirubicin, Etoposide, Fludarabine, Idarubicin, Mitoxantrone, Paclitaxel, Vinblastine, Vindesine, Vinorelbine	Overexpression of P-glycoprotein 1
**LiKa**	Nalm-6	Daunorubicin	Colchicine, Dexamethasone, Doxorubicin, Epirubicin, Etoposide, Fludarabine, Idarubicin, Mitoxantrone, Paclitaxel, Prednisolone, Vinblastine, Vincristine, Vindesine, Vinorelbine	Overexpression of P-glycoprotein 1
**NaKu**	Nalm-6	Prednisolone	4-OH Cyclophosphamide, Carboplatin, Cisplatin, Cladribine, Colchicine, Cytarabine, Daunorubicin, Dexamethasone, Doxorubicin, Epirubicin, Etoposide, Idarubicin, Ifosfamide, Oxaliplatin, Vindesine	Downregulation of FLT-3
**MaKo**	Nalm-6	Cytarabine	4-OH Cyclophosphamide, Cladribine, Clofarabine, Vincristine	Downregulation of FOXI-1
**BiBo**	BJAB mock	Vincristine	4-OH Cyclophosphamide, Colchicine, Cytarabine, Daunorubicin, Paclitaxel, Vinblastine, Vindesine, Vinorelbine	Overexpression of Bcl-2
**7CCA**	BJAB mock	Doxorubicin	Cisplatin, Cladribine, Clofarabine, Colchicine, Cytarabine, Daunorubicin, Epirubicin, Etoposid, Fludarabine, Fluorouracil, Idarubicin, Mitoxantrone, Oxaliplatin, Paclitaxel, Vinblastine, Vincristine, Vindesine, Vinorelbine	Downregulation of Caspase-3
**NiWi-Dau**	K562	Daunorubicin	Colchicine, Doxorubicin, Epirubicin, Idarubicin, Mitoxantrone, Paclitaxel, Prednisolone, Vinblastine, Vincristine, Vindesine, Vinorelbine	Downregulation of Harakiri
